# Cognitive impairment and exploitation: connecting fragments of a bigger picture through data

**DOI:** 10.1093/pubmed/fdae266

**Published:** 2024-10-02

**Authors:** Aisha M Abubakar, Rowland G Seymour, Alison Gardner, Imogen Lambert, Rachel Fyson, Nicola Wright

**Affiliations:** Rights Lab, University of Nottingham, University Park, Nottingham NG7 2RD, UK; School of Mathematics, University of Birmingham, Birmingham B15 2TT, UK; Rights Lab, University of Nottingham, University Park, Nottingham NG7 2RD, UK; School of Sociology and Social Policy, University of Nottingham, University Park, Nottingham NG7 2RD, UK; Rights Lab, University of Nottingham, University Park, Nottingham NG7 2RD, UK; School of Sociology and Social Policy, University of Nottingham, University Park, Nottingham NG7 2RD, UK; Rights Lab, University of Nottingham, University Park, Nottingham NG7 2RD, UK; School of Health Sciences, Queens Medical Centre, University of Nottingham, Nottingham NG7 2UH, UK

**Keywords:** adults, cognitive impairment, disabilities, England, exploitation, learning, mental health, modern slavery, safeguarding, services

## Abstract

**Background:**

Exploitation poses a significant public health concern. This paper highlights ‘jigsaw pieces’ of statistical evidence, indicating cognitive impairment as a pre- or co-existing factor in exploitation.

**Methods:**

We reviewed English Safeguarding Adults Collection (SAC) data and Safeguarding Adults Reviews (SARs) from 2017 to 22. Data relevant to exploitation and cognitive impairment were analysed using summary statistics and ‘analysis of variance’.

**Results:**

Despite estimates suggesting cognitive impairments may be prevalent among people experiencing exploitation in England, national datasets miss opportunities to illuminate this issue. Although SAC data include statistics on support needs and various forms of abuse and exploitation, they lack intersectional data. Significant regional variations in recorded safeguarding investigations and potential conflation between abuse and exploitation also suggest data inconsistencies. Increased safeguarding investigations for people who were not previously in contact with services indicate that adults may be ‘slipping through the net’. SARs, although representing serious cases, provide stronger evidence linking cognitive impairment with risks of exploitation.

**Conclusions:**

This study identifies opportunities to collect detailed information on cognitive impairment and exploitation. The extremely limited quantitative evidence-base could be enhanced using existing data channels to build a more robust picture, as well as improve prevention, identification and response efforts for ‘at-risk’ adults.

## Introduction

The advent of big data, machine learning and artificial intelligence has increased emphasis on using data to inform decision-making in public and social policy.^1^ This ‘datafication’ of policy is being applied to anti-poverty programmes,^2^ welfare states in developed countries^3^ and children’s social care.^4^ To develop effective policies based on data, however, there needs to be sufficient high-quality data available.

In recent years, modern slavery, human trafficking and wider forms of exploitation, such as ‘cuckooing’ (a form of criminal exploitation where a victim’s home is coercively taken over for criminal activities),^5^ are issues that have gained increasing recognition as a public health challenge.^6^^,^^7^ Yet, data on the scale of the issue and the characteristics of those affected remain limited. This paper reviews current data on the intersection between cognitive impairment and exploitation in England to evaluate the extent to which they can be used to inform policy. Specifically, our research asks which datasets contain information about both exploitation and cognitive impairment, and what strengths and limitations characterize available data? What trends can be quantified to understand how people with cognitive impairments are being exploited?

In this study, we define the term ‘cognitive impairment’ broadly to include learning disabilities, mental illnesses or other psychosocial impairments that affect processing, understanding and memory, and therefore may cause additional challenges in everyday life.^8^ We used this framing because we recognize that although these categories are distinct, they may have similar social impacts in terms of functioning and relationships. Such conditions are not always readily distinguished for recording purposes from a practitioner perspective; hence our study tries to work from this angle.

Exploitation refers to using someone’s vulnerability for one’s own gain or profit.^9^ The exploitation of individuals with cognitive impairment can take many forms, including financial, sexual and criminal exploitation.^10^^,^^11^ It may also include experiences of ‘modern slavery’ encompassing human trafficking, organ harvesting, debt bondage, domestic servitude, forced labour and forced/early marriage.^12^^,^^13^ ‘Mate crime’, a hidden and underreported phenomenon involving ‘exploitative familiarity’, encompasses various forms of exploitation where people pretend to be friends to exploit others.^14^ Again, a broad definition was adopted to gain an overview of the spectrum of exploitation known to be facing people with cognitive impairment, not limited solely to modern slavery and human trafficking.

The presence of a connection between exploitation and cognitive impairment in the UK has been suggested by a handful of studies. A recent pilot study in Nottingham analysing a unique dataset from the local authority’s slavery and exploitation team found that 31% of referrals for support involved individuals with diagnosed cognitive impairments.^5^ Studies focussing on the elderly have also identified cognitive impairment and psychological well-being as risk factors for abuse, including financial exploitation.^15^^,^^16^ Moreover, learning disability has been highlighted as a risk factor for modern slavery and human trafficking,^17–19^ while criminal exploitation often targets those with mental health needs, learning disabilities or substance misuse issues, sometimes manifesting as ‘cuckooing’.^20^ In one large-scale modern slavery case, 68% (of 60 victims) had substance misuse issues, and 20% had learning disabilities or mental health needs, with 62% of victims not receiving any support from services.^21^ Additionally, varied forms of cognitive impairment experienced by victims often co-exist with, or are exacerbated by environmental factors and life trauma, such as poverty and childhood abuse.^22–24^ A forthcoming scoping review found that many relevant studies focus on children, leaving a gap in research and data on adults at risk.^25^ Furthermore, studies often concentrate on specific forms of modern slavery and exploitation,^25^ contributing to a fragmented evidence base.

Existing statistical sources on exploitation often do not record cognitive impairment, leading to a lack of data concerning the way cognitive impairment may intersect with other social issues for effective planning and policy interventions.^26^ For example, the National Referral Mechanism (NRM) provides support to suspected victims of modern slavery and holds data on those referred, but due to a high bar for referral and the fact that many people do not consent to enter the service, it presents only a partial view of the numbers and characteristics of modern slavery survivors.^27–29^ Additionally the NRM publishes no data on disability. This stands in contrast to existing data on domestic abuse. For example, ‘SafeLives’, the largest national dataset on individuals accessing support for domestic abuse, records forms of disability encountered in cases of diverse forms of abuse.^30^

Research and data in the UK context therefore remain fragmented, forming pieces of a jigsaw that are yet to be pieced together. This data gap is a significant public health concern, as evidence suggests that people with a disability are more likely to experience various forms of abuse and exploitation, particularly if their health condition is ‘hidden’.^10^^,^^31–33^ Our study explored what could be contributed by combining existing local and national data sources, as well as the limitations of these datasets.

## Methods

We began by examining three potential data sources for national estimates on cognitive impairment and exploitation including NRM statistics, the Crime Survey for England and Wales (CSEW) and Family Resources Survey (FRS). However, each of these datasets either neglected to publish data on disability, or, if these data were present, did not include information on exploitation. See Appendix A1 in the Online Supplementary Material for a more detailed description of these datasets, including their strengths and limitations.

We then identified two data sources containing information about disability and exploitation, collected at the local authority level. These include the Safeguarding Adults Collection (SAC) and Safeguarding Adults Reviews (SARs).

### Safeguarding adults collection

Since 2010, English local authorities or Councils with Adult Social Services Responsibilities have been mandated to report statistics on vulnerable individuals aged 18 or over at risk of abuse, neglect or exploitation. This aims to ensure the safety and well-being of adults with care and support needs, and to prevent and respond to maltreatment.

Section 42 (s.42 hereafter) of the 2014 Care Act requires local authorities to investigate when they have reasonable grounds to suspect that an adult with care and support needs is experiencing or is at risk of experiencing abuse, neglect or exploitation. Investigations collate information about the adult and their circumstances, assess risks to their safety and determine the best way to protect them. The SAC aggregates data on s.42 investigations, which are published by National Health Service (NHS) Digital. Since 2017, SAC has included cases of modern slavery and other types of exploitation in its statistics, and therefore we focussed on the period covering 2017–18 to 2021–22 (data relates to 1 April to 31 March of the following year). See Appendix A2 for more details on the SAC.

However, SAC does not publish data on the intersection between adults with specific care and support needs and experiences of exploitation. We therefore supplement SAC data with microlevel evidence extracted from SARs featuring exploitation during the same period (2017–22).

### Safeguarding adults reviews

SARs are commissioned in cases where an adult with care and support needs has suffered serious harm. We reviewed SARs within a library published by the National Network for Chairs of Safeguarding Adults Boards, selecting all with references to exploitation. Our final sample included 58 reviews, covering 71 individuals with confirmed or suspected cases of exploitation. Appendix A3 outlines the search protocol, inclusion criteria and the process leading to the final sample. Systematic data extraction used Qualtrics to numerically code and gather details on the recorded forms of exploitation and health conditions. All identified SARs were screened by one reviewer, with a second reviewer checking 20% or 10 returns (whichever was lower).

### Analytical approach

Our analysis relies mainly on administrative data, which is mostly available as text-based or in aggregated formats such as counts or percentages. We found that the data, or its log transformation, was largely symmetric, unimodal and not skewed. Therefore, descriptive statistics considered the mean, variance, counts and percentages to identify typical values and measure variations in the dataset.

Given that s.42 enquiries are mandated by national legislation and produced at the local authority level, we expect the variance between authorities and regions to be consistent. Hence, we used analysis of variance (ANOVA) to examine statistical differences in the log-transformed number of s.42 enquiries over time and between English regions, with the assumption that the data in each year and region are independent. To mitigate any potential concerns about dependence and heteroscedasticity (i.e. inconsistent standard deviations over time), we excluded the first two years from when s.42 enquiries were being introduced and analysed the data at a regional level to harmonize any potential differences in local authority level decision-making processes. We used 5% as the statistical level of significance, except where effects were not statistically significant, we checked up to 10%.

## Results

### Rising numbers of Section 42 investigations mask significant regional variations

Population-adjusted national estimates of safeguarding enquiries show a rise in both safeguarding concerns and enquiries per 100 000 people between 2017–18 and 2021–22 (Appendix B1), but statistical analysis suggests no significant year-on-year differences in means over the 5-year period at the 10% level (ANOVA, *F*_(4,746)_ = 1.78; *P* = 0.1310).

**Fig. 1:**
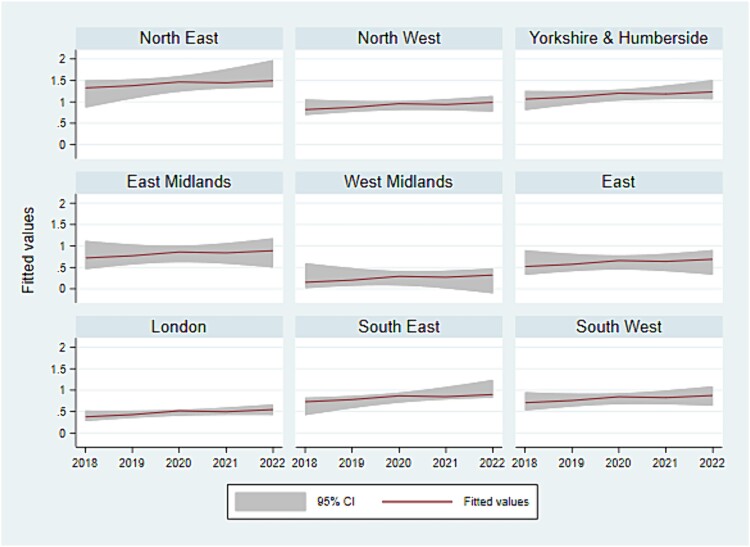
Population-adjusted trends in s.42 enquiries, regional ANOVA. Source: Authors' estimations using SAC data (2017–18 to 2021–22).

However, Fig. 1 reveals statistically significant differences in average counts of s.42 enquiries across regions (ANOVA, *F*_(8,731)_ = 27.35; *P* = 1 × 10^−3^) and overtime (ANOVA, *F*_(4,731)_ = 2.08; *P* = 0.082), with pairwise comparisons of means, indicating that the North East had significantly higher average counts than most regions, except the Yorkshire & Humberside where differences were not statistically different. The West Midlands, East and London had significantly lower counts than the East Midlands, North West, Yorkshire & Humberside, South East and South West. See detailed statistical outputs in Appendix Table B1.

Previous research suggests that context-specific factors such as changes in organizational structure, differences in standard processes and reporting procedures may influence the number of enquiries reported within regions and across local authorities.^34^ For example, two local authorities (outliers) noted changes in the decision-making process and the implementation of a new data capture system in the 2022 collection.^35^  Appendix B2 provides further explanations for these regional level differences.

### Increasing safeguarding investigations for adults previously unknown to services

**Fig. 2:**
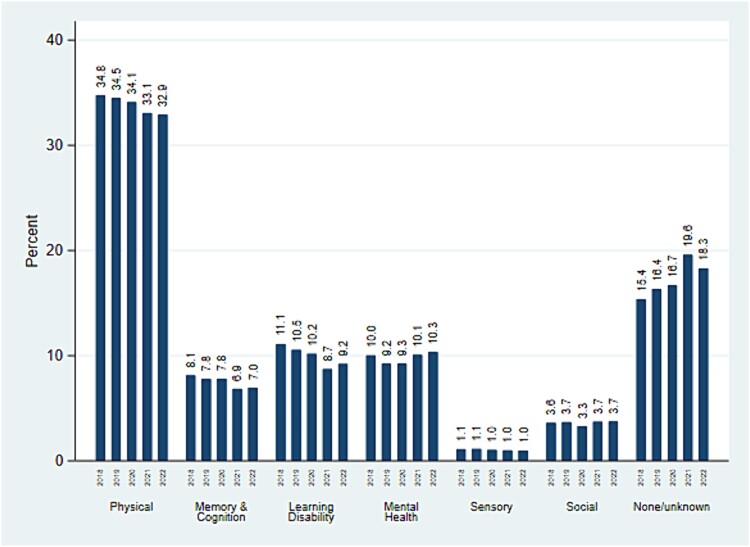
Primary support reason as a percentage of people involved in s.42 enquiries. Source: Authors' estimations using SAC data (2017–18 to 2021–22).

Next, we plot the national trend in safeguarding enquiries by type of primary support reason. Figure 2 indicates that people with mental health vulnerabilities and learning disability support needs are most consistently featured in safeguarding investigations compared with those with memory support needs, and we find a declining trend in enquiries involving those in receipt of memory or learning disability support. It is unclear whether this results from fewer adults receiving support for these issues, or alternative factors such as fewer people reporting these disabilities;^36^ however, there is an increase in safeguarding enquiries for people with no or unknown previous support, particularly in 2021 when coronavirus disease 2019 (COVID-19) lockdowns are likely to have affected these numbers. This implies individuals with support needs may be ‘slipping through the net’ until a serious incident occurs, possibly connected to higher barriers for access to services driven by funding reductions.^37^^,^^38^

### Conflating exploitation with abuse

The annual changes in the number of completed safeguarding investigations mirror those of ongoing investigations, with estimates peaking at 2020 (Table 1).

**Table 1:** Type of risk as a percentage of the total number of concluded s.42 enquiries

	2017–18	2018–19	2019–20	2020–21	2021–22
Concluded s.42 enquiries	119 100	125 365	150 455	149 540	147 930
Concluded s.42/100 000 people	214	224	267	264	262
***Abuse:***					
Physical abuse	34 350 [28.8%]	37 630 [30.0%]	42 340 [28.1%]	40 240 [26.9%]	39 000 [26.4%]
Sexual abuse	6645 [5.6%]	6920 [5.5%]	7685 [5.1%]	7410 [5.0%]	7295 [4.9%]
Psychological abuse	20 210 [17.0%]	23 480 [18.7%]	28 535 [19.0%]	30 080 [20.1%]	28 280 [19.1%]
Discriminatory abuse	870 [0.7%]	980 [0.8%]	1155 [0.8%]	1395 [0.9%]	2320 [1.6%]
Organizational abuse	6425 [5.4%]	7040 [5.6%]	8810 [5.9%]	8920 [6.0%]	11 760 [7.9%]
Neglect/omission	49 695 [41.7%]	54 050 [43.1%]	65 590 [43.6%]	61 190 [40.9%]	64 330 [43.5%]
Domestic abuse	6365 [5.3%]	7990 [6.4%]	10 825 [7.2%]	13 880 [9.3%]	13 035 [8.8%]
Self-neglect	6435 [5.4%]	7790 [6.2%]	10 245 [6.8%]	12 920 [8.6%]	13 990 [9.5%]
***Exploitation:***					
Financial abuse/exploitation	22 565 [18.9%]	24 625 [19.6%]	29 180 [19.4%]	28 225 [18.9%]	26 130 [17.7%]
Sexual exploitation	890 [0.7%]	1060 [0.8%]	1260 [0.8%]	1665 [1.1%]	1235 [0.8%]
Modern slavery	245 [0.2%]	340 [0.3%]	480 [0.3%]	525 [0.4%]	545 [0.4%]

Note: Authors' estimations using SAC data (2017–18 to 2021–22). The percentages of concluded enquiries by type of risks are weighted by the total number of concluded s.42 investigations. Multiple types of risks can be logged per concluded s.42 enquiry. As a result, the total percentage across all types of risks can sum up to a value higher than 100 in each period. Some local authorities apply only one type of risk to each enquiry, while others apply as many as are applicable, hence these data should be treated with caution.

When examining the prevalence of various types of abuse and exploitation in concluded safeguarding enquiries, trends in Table 1 indicate a decreasing proportion of completed s.42 cases involving sexual and financial abuse/exploitation, while domestic abuse cases have shown a gradual increase overtime.^39^^,^^40^ Modern slavery, although a small proportion, more than doubled from 245 concluded investigations to 545 from 2018 to 22, probably reflecting increasing awareness among frontline practitioners.

Again, differing reporting practices across local authorities may be impacting identification of exploitation. Low returns on sexual exploitation potentially represent conflations between exploitation and abuse by local authorities, as some do not collect information on sexual exploitation as a separate category.^35^ Additionally, exploitation by a family member (e.g. forced marriage) can be recorded under domestic abuse,^41^ obscuring the identification and protection of adults experiencing this form of modern slavery. There are also links between exploitation and neglect/acts of omission.^42^

### Almost all SARs featuring exploitation document a mental health condition or cognitive disorder, with individuals experiencing multiple forms of exploitation

We now use microlevel evidence from SARs to better understand intersections between exploitation and cognitive impairment. Recent SARs studies reveal that most adults experiencing ‘homelessness’ or ‘self-neglect’ exhibit mental health or learning disabilities,^43^^,^^44^ while another study found 13 SARs involving sexual exploitation of children with complex needs.^42^

The health profile of individuals in the SARs that we examined indicates that almost all (96%) of the individuals experiencing exploitation had pre- or co-existing cognitive or mental health disorders, see Appendix C (Table C1, Panel A). Approximately 81% of individuals have mental health conditions, others have intellectual disabilities (24%), autism spectrum or attention deficit hyperactivity disorder (12%), brain injury (10%) or memory disorders (9%), with these conditions often co-occurring (Appendix Table C1, Panel B).

Moreover, many individuals featuring in SARs were subject to more than one form of exploitation (Table 2). Financial exploitation (74%) emerges as the most widespread, followed notably by criminal exploitation (37%), mate crime (35%) and sexual exploitation (27%). Cuckooing was the most common form of criminal exploitation, while instances of human trafficking (12%) and labour exploitation (10%) were reported less frequently.

**Table 2:** Forms of exploitation featured in SARs

	%	Frequency
Sexual	26.5	18
Financial	73.5	50
Mate crime	35	24
Criminal:	37	37
*Cuckooing*	[76]	19
*Other (e.g. drug dealing)*	[12]	3
*Cuckooing and other*	[12]	3
Trafficking	11.8	8
Labour	10.3	7
Unspecified/unknown	8.8	6

Note**:** Authors’ estimations using 58 SARs where exploitation was a factor, covering 68 (out of 71) individuals with cognitive impairment. People may experience multiple forms of exploitation, which results in a total value greater than 100%. The estimated share of criminal exploitation (37%) covers individuals who may have experienced at least one type of criminal exploitation including cuckooing or any other criminal exploitation. Mate crime may involve sexual, criminal, labour exploitation or human trafficking.

Mate crime and human trafficking often overlapped with other categories (Appendix C2). Notably, 70% of individuals described as experiencing ‘mate crime’ experienced either financial (38%) or both financial and criminal exploitation (33%).

SARs document a small proportion of enquiries, suggesting that instances of exploitation identified in SARs represent only a fraction of a wider problem. This analysis therefore underscores that many more individuals with cognitive impairments may be at risk of exploitation, with long-lasting consequences for their health and quality of life.

## Discussion

### Main finding of this study

Although existing data are sparse, objective statistical evaluation methods reveal a demonstrable intersection between exploitation and cognitive impairment, which existing national data collection instruments fail to expose. SAC data on this issue are limited by local variations in recording and initiating safeguarding enquiries and the conflation of abuse and exploitation. Such variation in data collection means that national policy on safeguarding adults may not be fully informed by a comprehensive understanding of exploitation prevalence. Although we acknowledge that these areas are complex, clearer guidelines could potentially yield valuable data to assist in preventing and responding to exploitation.

We find that people with mental health vulnerabilities or learning disability needs are most consistently featured in both safeguarding investigations and actual or suspected cases of exploitation compared with those with memory support needs. Despite this, significant cuts to mental health services in England leave many without support, raising concerns about potential harm to individuals,^45^ especially with the discontinuation of the 10-year mental health plan at the policy level. Those with mild cognitive impairments and those without access to support services may be failing to be identified until a point of crisis.

### What is already known on this topic

Many studies focus on mental health consequences arising from exploitation, while only a handful of papers have considered cognitive impairment as a vulnerability to exploitation in adults. A scoping review found limited evidence, with relevant studies often focusing on children and sexual exploitation.^46^ Despite limited research and gaps in quantitative data, our analysis suggests that adults with cognitive impairments are experiencing exploitation. This therefore underscores the need for further research into how a broader spectrum of cognitive conditions influence vulnerabilities to exploitation.

### What this study adds

This is the first study, to our knowledge, that identified potential opportunities to collect more detailed information on cognitive impairment and exploitation through existing data collection pathways. SAC data identify a growing trend in safeguarding investigations and the potential for differences in local processes explaining the observed variations in s.42 enquiries. Importantly, the analysis of SARs provides tentative evidence on the high co-occurrence of impairments and exploitation among the most serious cases of harm.

Despite the limitations of existing datasets, we offer three important data recommendations. *First*, this paper underscores the importance of intersectional data on disability and exploitation. We emphasize the need to integrate disability information into NRM statistics, while the incorporation of exploitation data to the FRS and CSEW could also advance research in this nascent area. *Secondly*, there is a need for coherence and consensus among local authorities in defining, identifying and recording abuse versus exploitation in the SAC and SARs, noting the interchangeability of these terms and the potential impact on effective investigation and prosecution. *Finally*, future analysis could examine the vulnerability of individuals with cognitive impairment to specific forms of exploitation, exploring relationships through regression analysis.

### Limitations of this study

SAC data contain limited number of exploitation types and do not explicitly specify what proportion of people with cognitive impairment had experienced exploitation. ANOVA assumed that SAC data are homoscedastic at the regional level even though local level processes may vary. SARs represent serious cases of exploitation with varying levels of detail. Some SARs may be missing from the library and many cases are not examined through SARs. We acknowledge that these administrative datasets have limitations that may potentially affect the generalizability of our findings, which are well described in other studies.^34^^,^^47^ Although the data we discussed relate to England, the datafication issue we highlight here has also been observed in wider UK and international contexts.^10^^,^^48^ Hence, improving the precision of data collection and collating/publishing intersectional data may be more widely applicable.

## Conclusions

This study reveals a critical intersection between cognitive impairment and exploitation, highlighting gaps in national data that fail to capture the full extent of this issue. To enhance prevention, identification and response efforts for ‘at-risk’ adults, we recommend integrating detailed disability data into existing datasets on modern slavery and exploitation, standardizing definitions and recording practices, and further research on specific vulnerabilities to different forms of exploitation. Addressing these gaps will be crucial for informed public health interventions and policymaking aimed at protecting vulnerable populations.

## Supplementary Material

Supplementary_Online_Appendix_fdae266

## Data Availability

The Safeguarding Adults Collection is available to download from NHS England Digital. Safeguarding Adults Reviews are available to download from the SAR library published by the National Network for Chairs of Adult Safeguarding Boards. The coded data or metadata used in the analysis of SARs are available from the authors upon reasonable request.

## Notes

**Aisha M. Abubakar**, Research Fellow in Cognitive Impairment and Exploitation (quantitative)

**Rowland G. Seymour,** Assistant Professor in Mathematics

**Alison Gardner,** Associate Professor in Public Policy and Administration

**Imogen Lamber,** Research Fellow in Cognitive Impairment and Exploitation (qualitative)

**Rachel Fyson**, Professor in Sociology and Social Policy

**Nicola Wright,** Associate Professor in Mental Health
