# Supplementary material for: Cognitive impairment and exploitation: connecting fragments of a bigger picture through data
Source: J Public Health (Oxf). 2024 Oct 2;46(4):498–505. doi: 10.1093/pubmed/fdae266 (PMC11638330; doi:10.1093/pubmed/fdae266)
Supplement: Supplementary_Online_Appendix_fdae266 [file supplementary_online_appendix_fdae266.pdf]

Cognitive impairment and exploitation: connecting fragments of a bigger picture through data

Online Supplementary Material

Appendix A. Details on data

A1. Strengths and limitations of potential data sources for national estimates

Table A1 provides a concise summary of potential data collection avenues for national research on exploitation, including the NRM, the CSEW, and the FRS. These routinely collected data sources present an excellent opportunity to gather information that can facilitate precise estimations of exploitation rates among people with cognitive impairments at the national level.

Table A1: Potential data sources for intersectional data on cognitive impairment and exploitation

Data source	Strengths	Limitations
National Referral Mechanism (NRM) Statistics	Contains data on worst forms of exploitation (modern slavery)	No data on health status and covers a heterogenous sample of individuals. Not designated as national statistics
Crime Survey for England and Wales (CSEW)	Provides information on impairment and abuse	Does not allow for the extrapolation of incidents of abuse into the category of exploitation.
Family Resources Survey (FRS)	Contains data on impairment types	No questions on the experiences of exploitation

The NRM is a framework for identifying and referring potential victims of modern slavery in the UK, as well as ensuring they receive the appropriate support. National estimates on modern slavery referrals via the NRM can be used to estimate exploitation prevalence in England. NRM statistics, while providing breakdowns by gender, age and nationality, do not include health status data, making it challenging for this study to explore intersections between cognitive impairment and exploitation using this data. Moreover, the NRM covers applicants from various nationalities, often without UK residency status, capturing a population different from other survey instruments. This divergence makes estimating the prevalence of exploitation in the British population using the NRM problematic, and many cases, especially among the British population, may remain hidden and unreported.

The CSEW estimates that between 2014 and 2020, people with cognitive impairment aged between 16 and 59 were more likely to be victims of different forms of domestic abuse and sexual assaults than people with other forms of impairment, particularly women.[1] While this may also extend to exploitation, it is not possible to extrapolate what incidents of abuse constitute exploitation in the CSEW.

The FRS is an annual survey that collects detailed information on living standards and circumstances of people in the UK, including self-reported disability status. While the FRS provides information on

impairment types, from which we could estimate the incidence of cognitive-related disabilities, it does not provide data on experiences of exploitation.

A2. Safeguarding Adults Collection (SAC)

Social workers, health professionals, the police, and other relevant stakeholders are all involved in safeguarding investigations under Section 42 (s.42) of the Care Act 2014. NHS Digital publishes SAC data, which includes the number of safeguarding concerns and s.42 enquiries, primary support needs of individuals and, inter alia, a breakdown of concluded s.42 enquiries by abuse or exploitation type. The SAC also provides data on s.42 enquiries reported by two categories of individuals with specific cognitive health conditions – those with learning, developmental, or intellectual disabilities. These are divided into two groups based on severity, with the first group comprising individuals with more severe conditions (including low functioning autism), and the second comprising a milder group, such as those with Asperger's syndrome or high-functioning autism. It is important to clarify that people with high functioning autism may not necessarily fall under the framework of learning, developmental or intellectual disabilities.

The geographical granularity of SAC data covers national, regional, and local authority levels annually, involving statistics from 152 CASSRs. However, due to a cyber-attack, the London Borough of Hackney could not submit data for the 2021 and 2022 returns, while the Isles of Scilly reported zero s.42 cases in 2022, resulting in a local authority sample of 150. We analysed data covering the period 2017/18 to 2021/22 in STATA 18, retrieved from NHS Digital.[2] While there are earlier rounds of the data, we focus on the period from 2017/18 onward when reporting on a wider range of exploitation types became mandatory, providing a trajectory of eleven types of abuse and exploitation contained in the data. To account for population size variations, demographic data from the 2021 Census was extracted from the Office for National Statistics (ONS) via NOMIS.[3]

While the SAC contains data on care and support needs, and specific types of exploitation including modern slavery, sexual, and financial exploitation, there are some limitations.

- Limited number of exploitation types are listed in the SAC.
- Quite generic and does not explicitly specify what proportion of people with cognitive impairment had experienced exploitation.
- Potential conflation of exploitation with abuse: The SAC may obscure certain types of exploitation such as, conflating financial abuse and exploitation, while modern slavery may cover a wide range of exploitation types.

A3. Safeguarding Adults Reviews (SARs)

SARs, conducted under Section 44 of the 2014 Care Act, are initiated by Safeguarding Adults Boards in cases where an adult with care and support needs has suffered serious harm or death, and abuse or exploitation is suspected. Formerly known as 'Serious Case Reviews', these assessments aim to uncover valuable lessons from particularly severe cases, contributing to the improvement of the safeguarding system for adults in vulnerable circumstances in England. These reviews were downloaded from the National Library of SARs.[4]

The initial criteria was based on three broad terms "exploit", "traffick", "slavery", which returned 171 documents (Table A2). Filtering out documents beyond our time scope (N=12), miscellaneous documents not fitting safeguarding adult reviews (N=14), and duplicates (N=35), we arrived at 100 documents for initial skim-reading in the second stage. During this phase, we excluded documents

where conceptual terms were present but not indicative of actual or suspected exploitation. Our focus on exploitation led to a final sample of 58 reviews eligible for inclusion in the study, comprising 47 individual case reports, 6 thematic reviews covering between 3 to 10 individuals each, along with 4 executive summaries and 1 learning brief. In total, the sample encompasses 71 individuals who experienced confirmed or suspected exploitation.

Table A2: Safeguarding adult reviews, final sample and inclusion criteria

**Panel A: Inclusion criteria**	
Included if "exploit"	136
Included if "slavery"	24
Included if "traffick"	11
Total eligible based on keywords:	***171***
**Panel B: First stage eligibility criteria**	
Excluded if 2016 + earlier	19
Excluded if 2023	3
Excluded if miscellaneous (e.g., tool kits, knowledge briefings, guidance notes)	14
Excluded if duplicate	35
Total eligible if SAR year in circa 2017 to 2022:	***100***
**Panel C: Second stage eligibility criteria**	
Excluded if not exploited (skim read)	37
Excluded if not exploited (full text read)	5
Total eligible for inclusion (final)	***58***
**Panel D: Breakdown of final sample**	
Full reports	47
Thematic reviews	6
Executive summaries	4
Learning briefs	1

To ensure inter-rater reliability, all identified SARs were screened by the lead author, with another author checking 20% or 10 returns (whichever was lower). Additionally, a second author checked and verified the coding process in Qualtrics.

While data on impairment and exploitation can be extracted from SARs, there are some important limitations.

- Focuses on only serious cases, making the findings observed at the lowest bounds
- Contains varying levels of detail
- Potentials for more SARs available but not uploaded in the National Library of Safeguarding Adults Boards.
- Potential bias in data entry, and less feasible to extract if a very large number of relevant reviews are identified.

Appendix B. Trends in s.42 enquiries

B1. Rising safeguarding concerns and s.42 enquiries are likely driven by regional variations

Population-adjusted national estimates of safeguarding enquiries are reported in Figure B1. The blue bars and left axis represent the number of safeguarding concerns raised between 2018 and 2022, while the red line and right axis depict trends in s.42 enquiries. We find a consistent rise in both safeguarding concerns and enquiries per 100,000 people between 2018 and 2022, with the most substantial growth occurring from 2019 to 2020. This upsurge in 2020 is not driven by the COVID-19 pandemic because the data period ended before the lockdown really took hold.[5] While the availability of weekly data may provide additional insights, monthly data found that the rise in domestic and psychological abuse well before the pandemic may have contributed to the 2020 upsurge.[6]

Figure B1: Trends in safeguarding concerns and s.42 enquiries.

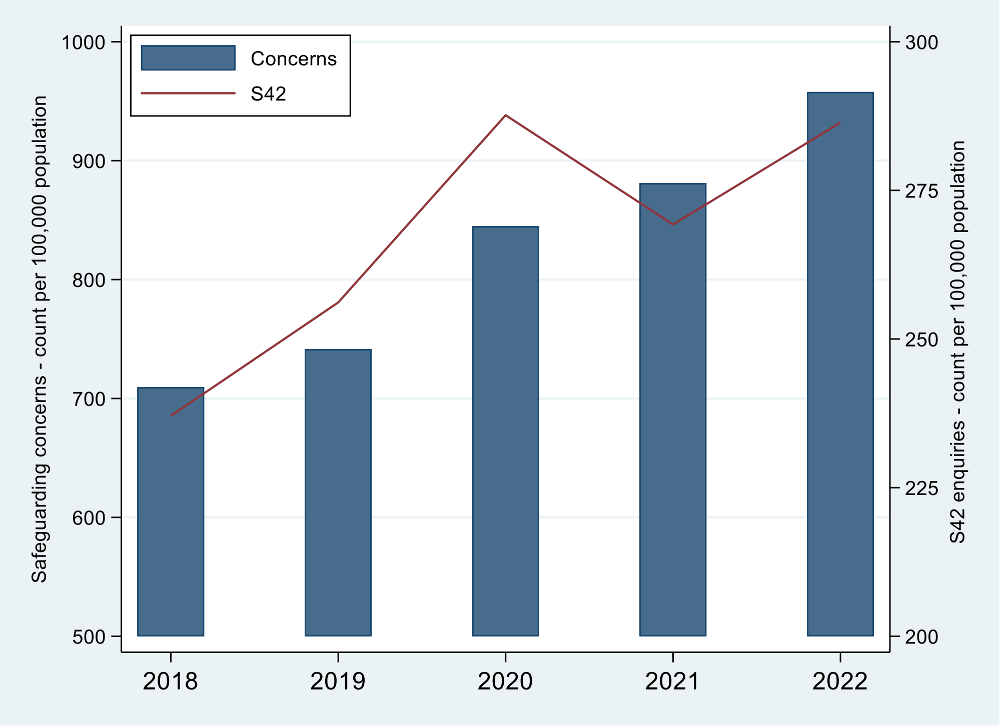

Next, an ANOVA was performed to statistically test the regional differences in s.42 enquiries (Table B1, Panel A). We find differences over time when regional differences are accounted for in the model. Additionally, Tukey's HSD Test for multiple comparisons found that the average counts of s.42 enquiries was significantly different between regions, indicated by darker shades in Table B1, Panel B).

When compared to the differences identified for other regions, the largest effect sizes (mean differences) are most significantly notable for the North East (between 0.51 and 1.17) and the Yorkshire & Humberside (between 0.34 and 0.91). These two regions have significantly higher counts of s.42 enquiries compared to other regions. We also find that the West Midlands, London, and East of England exhibit the lowest counts compared to other regions including the North West, East Midlands, South East and South West.

Table B1: Regional ANOVA and pairwise comparisons of means

**Panel A: ANOVA**					
	Partial Sum of Squares	df	Mean Square	F	P-Value
Model	77.159544	12	6.429962	18.93	0.000
Region	74.311977	8	9.288997	27.35	0.000
Time	2.8230919	4	0.705773	2.08	0.082
Residual	248.30997	731	0.339685		
Total	325.46952	743	0.438048		

**Panel B: Pairwise comparisons of means**					
				Tukey [95% conf. interval]	
	Mean Difference	Standard Error	P-value	Lower Bound	Upper Bound
North West vs North East	-0.506	0.093	0.000	-0.795	0.216
Yorkshire & Humberside vs North East	-0.262	0.101	0.192	-0.577	0.053
East Midlands vs North East	-0.606	0.116	0.000	-0.967	-0.245
West Midlands vs North East	-1.173	0.103	0.000	-1.493	-0.853
East vs North East	-0.804	0.109	0.000	-1.144	-0.465
London vs North East	-0.948	0.088	0.000	-1.223	-0.674
South East vs North East	-0.597	0.097	0.000	-0.898	-0.296
South West vs North East	-0.623	0.103	0.000	-0.944	-0.302
Yorkshire & Humberside vs North West	0.244	0.087	0.115	-0.026	0.513
East Midlands vs North West	-0.100	0.104	0.989	-0.423	0.222
West Midlands vs North West	-0.667	0.089	0.000	-0.943	-0.391
East vs North West	-0.298	0.096	0.050	-0.596	0.000
London vs North West	-0.442	0.071	0.000	-0.664	-0.221
South East vs North West	-0.091	0.082	0.971	-0.345	0.162
South West vs North West	-0.117	0.089	0.928	-0.394	0.160
East Midlands vs Yorkshire & Humberside	-0.344	0.111	0.052	-0.689	0.002
West Midlands vs Yorkshire & Humberside	-0.910	0.097	0.000	-1.213	-0.608
East vs Yorkshire & Humberside	-0.542	0.104	0.000	-0.865	-0.219
London vs Yorkshire & Humberside	-0.686	0.082	0.000	-0.940	-0.432
South East vs Yorkshire & Humberside	-0.335	0.091	0.007	-0.617	-0.053
South West vs Yorkshire & Humberside	-0.360	0.098	0.007	-0.664	-0.057
West Midlands vs East Midlands	-0.567	0.112	0.000	-0.916	-0.217
East vs East Midlands	-0.198	0.118	0.762	-0.566	0.170
London vs East Midlands	-0.342	0.099	0.017	-0.651	-0.033
South East vs East Midlands	0.009	0.107	1.000	-0.324	0.342
South West vs East Midlands	-0.016	0.113	1.000	-0.367	0.334

East vs West Midlands	0.369	0.105	0.014	0.041	0.696
London vs West Midlands	0.224	0.084	0.154	-0.035	0.484
South East vs West Midlands	0.575	0.092	0.000	0.288	0.863
South West vs West Midlands	0.550	0.099	0.000	0.242	0.859
London vs East	-0.144	0.091	0.815	-0.428	0.139
South East vs East	0.207	0.099	0.488	-0.103	0.516
South West vs East	0.182	0.106	0.735	-0.147	0.510
South East vs London	0.351	0.076	0.000	0.115	0.587
South West vs London	0.326	0.084	0.004	0.065	0.587
South West vs South East	-0.025	0.093	1.000	-0.314	0.264

B2. Differences in local processes potentially drive regional variations

Although 95% of the distribution of s.42 enquiries in 2022 across local authorities fell within a range of 0 to 7 enquiries per 1000 people in 2022, two outliers stood out with 23 and 12 enquiries per 1000 people, respectively (Figure B2, left panel). These two local authorities respectively noted changes in the decision-making process and the implementation of a new data capture system,[7] which might have influenced the observed enquiry rates and the consistency of data compared to previous years. For this reason, a logarithmic transformation was applied to eliminate measurement errors and restore normality (Figure B2, right panel). Log transformation is a data transformation method which replaces each variable 'x' with a log(x), facilitating more robust statistical analyses.

Figure B2: Histograms of s.42 enquiries per 1000 people in local authorities, 2021-22.

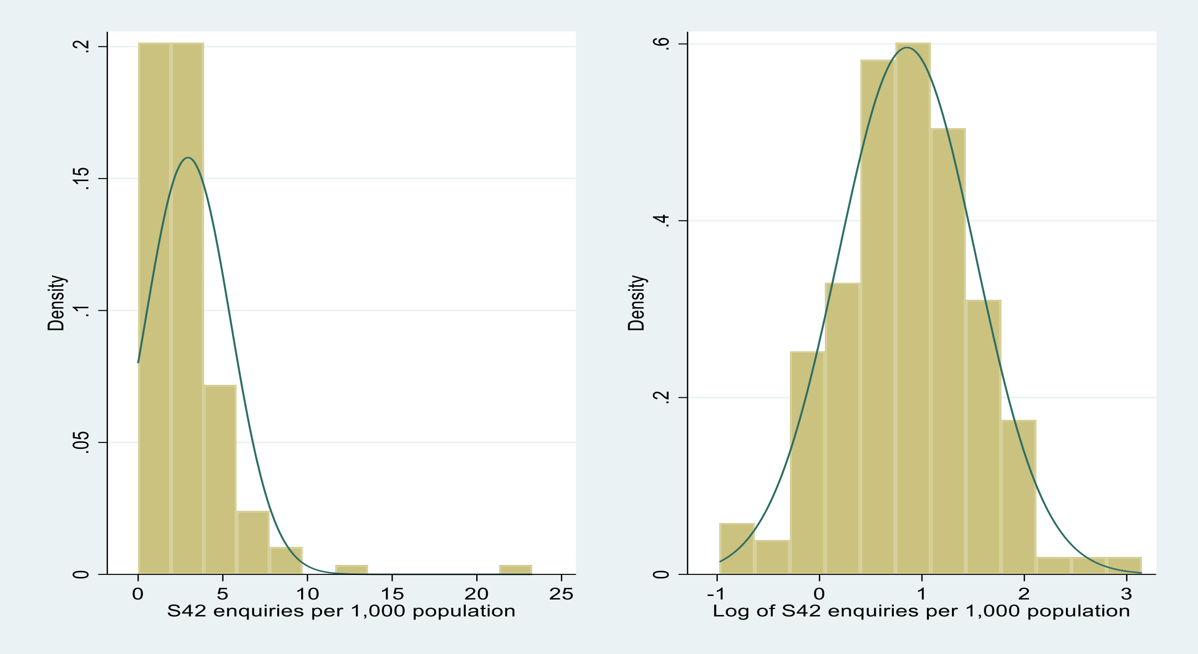

Furthermore, a survey of local definitions was commissioned in 2018 to ascertain how local authorities defined key elements of adult safeguarding activity in their 2017/18 SAC submission.[8] A total of 78 local authorities responded to the survey, representing 51% response rate. Table B2 provides a regional distribution of responses across these local authorities. Based on the question: "*Are there processes in place in your local authority that result in some safeguarding concerns being addressed before they*

reach the safeguarding team and therefore are not reported in the SAC?", responses can be used to aid the interpretation of any differences between local authorities.

Table B2: Response rate for the 2018 survey of local definitions

Region	Responded	Did not respond	Total
North East	9 (75%)	3 (25%)	12
North West	10 (43%)	13 (57%)	23
Yorkshire & Humberside	9 (60%)	6 (40%)	15
East Midlands	7 (78%)	2 (22%)	9
West Midlands	7 (50%)	7 (50%)	14
East	3 (27%)	8 (73%)	11
London	13 (39%)	20 (60%)	33
South East	7 (37%)	12 (63%)	19
South West	13 (81%)	3 (19%)	16
Total	78 (51%)	74 (49%)	152

A regional comparison of whether there are local processes in place that lead to some safeguarding concerns being addressed before they reach the safeguarding team and therefore, not reported in the SAC shows that out of the 78 local authorities that returned the 2018 survey of local definitions, just under half had processes in place. Indeed, Figure B3 suggests that local processes in recording safeguarding concerns may eventually determine the counts of s.42 enquiries and by implication, partly explain the regional variations in s.42 enquiries. Over time, local authorities that responded "yes" to having local processes in place have lower counts of s.42 enquiries compared to those who had no processes in place.

Figure B3: Variations in local procedures lead to variations in s.42 counts

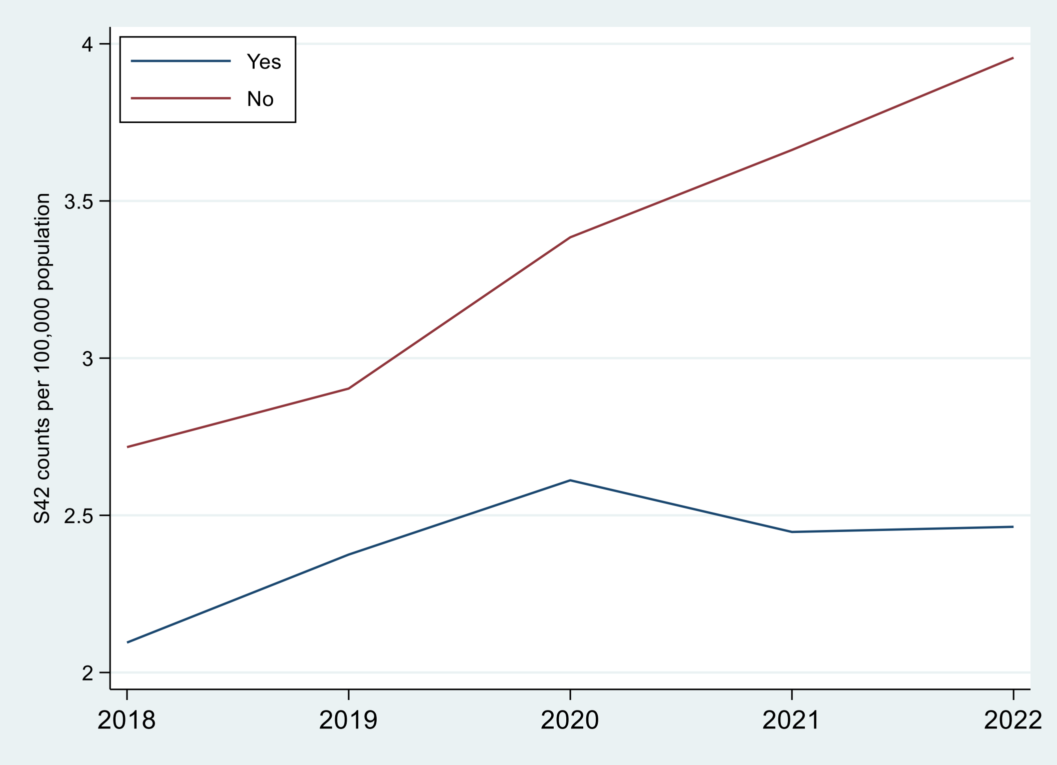

Regionally, Figure B4 shows that despite having the third highest incidence of s.42 enquiries, 8 out of 10 (80%) local authorities in the North West reported having procedures in place. In contrast, 5 out of 7 (71%) local authorities in the West Midlands who responded to the survey report not having processes in place, yet this region reports the lowest s.42 counts.

Figure B4: Safeguarding concerns are triaged before they reach the safeguarding team.

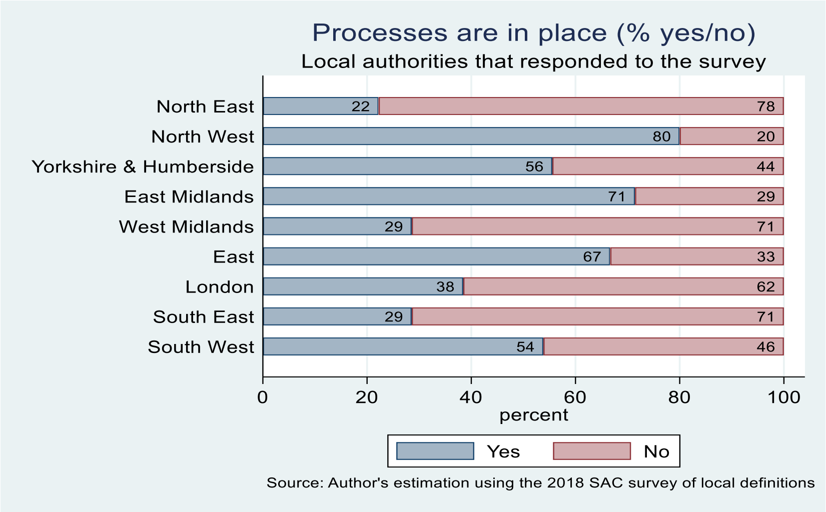

To put this into clearer perspective, Figure B5 indicates that very few regional variations occur in s.42 enquiry counts among the sample of local authorities that have local processes in place. Among local authorities that do not have processes in place, we see greater regional variations, with counts being highest in the North East and East Midlands regions compared to others. This finding therefore underscores the need for a harmonised data recording procedure across authorities and regions.

It should be noted that although the 2018 survey of local definitions only covers a specific period, we acknowledge the possibility of changes in local processes over time. Hence this analysis should be treated as a comparison for context, and not as a direct reflection of current processes.

Figure B5: Variations in local procedures lead to variations in s.42 counts, regional disaggregations

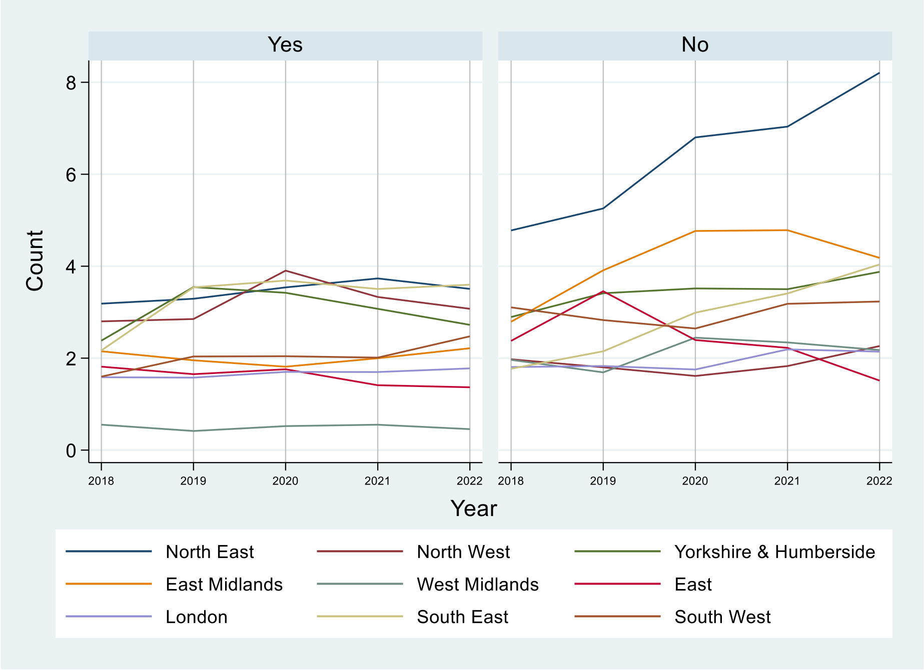

Appendix C. Health and exploitation profiles of individuals in SARs

C1. There is a demonstrable intersection between exploitation and different forms cognitive impairments

Table C1 (Panel A) indicates that different forms of cognitive impairment and mental health conditions are documented in 96% of safeguarding adults review cases, while only 3% exclusively have physical health needs or no impairment at all (1%). Furthermore, Table C1 (Panel B) illustrates that 81% of cases have mental health conditions, including anxiety and/or depression (44%), personality disorders (27%), schizophrenia (24%), and less commonly, post-traumatic stress disorder (PTSD, 13%). Other conditions that follow include intellectual disabilities (24%), brain injury (10%), autism spectrum or attention deficit hyperactivity disorder (ADHD, 12%), and memory disorders (9%). Furthermore, 77% of these individuals have substance misuse issues, while 50% have additional physical health needs. It is important to note that people may have multiple forms of cognitive impairment which results in a total value greater than 100% in Panel B.

Table C1: Health profile of individuals featuring in SARs

	%	Freq.
**Panel A: Types of health conditions (N=71)**		
Cognitive impairment	95.8	68
Physical impairment	2.8	2
No impairment	1.4	1
**Panel B: Types of health conditions, people cognitive impairment (N=68)**		
Intellectual disability	23.5	16
Autism spectrum or ADHD	12%	8
Memory problems	8.8	6
Brain injury	10.3	7
Mental health	81%	55
Anxiety and/or depressive disorder	[44%]	[24]
PTSD	[13%]	[7]
Personality disorder	[27%]	[15]
Schizophrenia	[24%]	[13]
Other (e.g., psychosis)	[26%]	[14]
Additional conditions:		
Substance misuse	76.5	52
Physical health needs	50.0	34
Note: Authors' estimations using 58 safeguarding adult reviews where exploitation was a factor.		

C2. Mate crime and trafficking were noted to overlap with various forms of exploitation

Figure C1 shows that among those who experienced trafficking, almost half experienced both financial and labour exploitation, with sexual and criminal exploitation also prevalent. Mate crime was closely linked to financial exploitation (38%) or a combination of financial and criminal exploitation (33%).

Figure C1: Forms of exploitation linked to trafficking or mate crime (%).

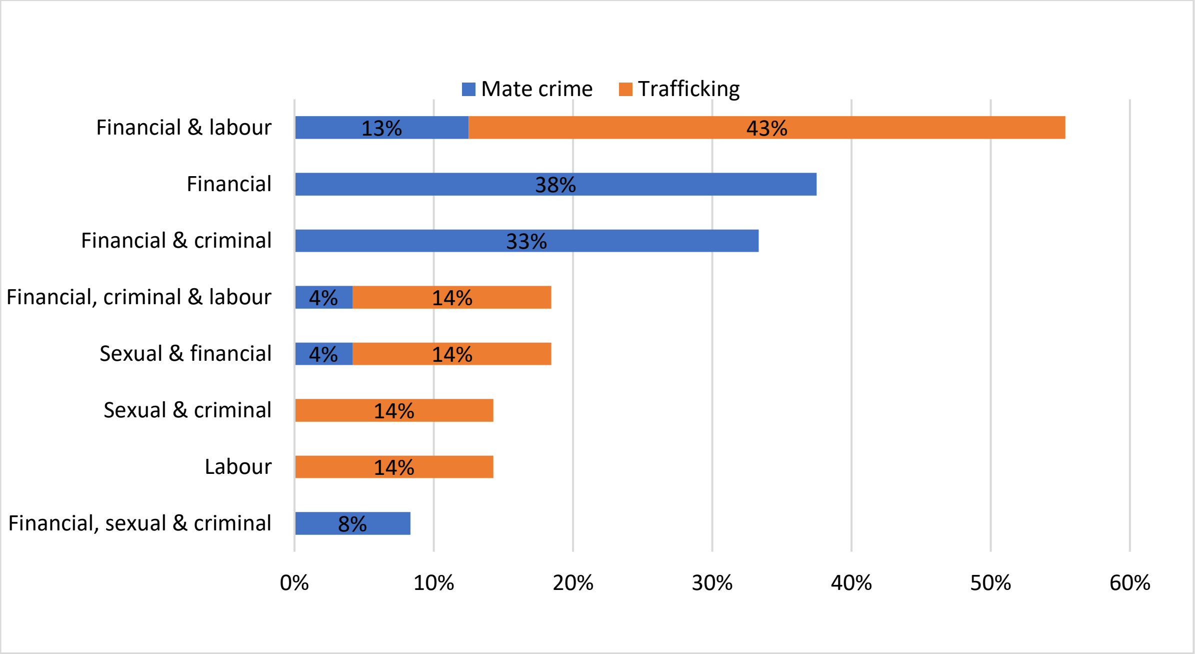

Additional references

1. ONS. *Outcomes for Disabled People in the UK: 2021. Disability and Crimes Reference Tables*. ONS website, article, released 10th Feb. 2022 2022; Available from: Retrieved 29th Nov. 2023 from https://www.ons.gov.uk/peoplepopulationandcommunity/healthandsocialcare/disability/articles/outcomesfordisabledpeopleintheuk/2021.
2. NHS Digital, *Safeguarding Adults Collection*. 2022, NHS Digital: Safeguarding Adults, England: Official Statistics: Retrieved 29th Nov. 2022 from https://digital.nhs.uk/data-and-information/publications/statistical/safeguarding-adults
3. ONS, *2021 Census - Demography and Migration*. 2022, Office for National Statistics: Official Census and Labour Market Statistics: Retrieved 11th Jan. 2023 from https://www.nomisweb.co.uk/.
4. National Network for Chairs of Adult Safeguarding Boards, *Safeguarding Adults Reviews (2017 to 2022)*. 2022, Safeguarding Adults Boards: Retrieved 25th Jun. 2023 from https://nationalnetwork.org.uk/search.html.
5. NHS Digital. *Safeguarding Adults, England, 2019-20*. 2020 [cited 2023 7th Jan. 2023]; Available from: https://digital.nhs.uk/data-and-information/publications/statistical/safeguarding-adults/2019-20.
6. LGA, *COVID-19 adult safeguarding insight project: findings and discussions.* 2020.
7. NHS Digital. *Safeguarding Adults, England, 2021-22*. 2022 [cited 2023 7th Jan. 2023]; Available from: https://digital.nhs.uk/data-and-information/publications/statistical/safeguarding-adults/2021-22.
8. NHS Digital, *Safeguarding Adults Collection, Survey of Local Definitions 2018*. 2018, NHS Digital: Retrieved 29th Nov. 2022 from https://digital.nhs.uk/data-and-information/find-data-and-publications/supplementary-information/2018-supplementary-information-files/safeguarding-adults-collection-survey-of-local-definitions-2018.
